# Transcriptional Regulation of Carnitine Catabolism in *Pseudomonas aeruginosa* by CdhR

**DOI:** 10.1128/mSphere.00480-17

**Published:** 2018-02-07

**Authors:** Jamie A. Meadows, Matthew J. Wargo

**Affiliations:** a Department of Microbiology and Molecular Genetics, University of Vermont Larner College of Medicine, Burlington, Vermont, USA; b The Vermont Lung Center, University of Vermont Larner College of Medicine, Burlington, Vermont, USA; Martin Luther University of Halle-Wittenberg

**Keywords:** metabolism, osmoprotectant, quaternary amine, transcriptional regulation

## Abstract

The common environmental bacterium and opportunistic pathogen *Pseudomonas aeruginosa* encodes diverse metabolic pathways and associated regulatory networks allowing it to thrive in these different environments. In an effort to understand *P. aeruginosa* metabolism and detection of host-derived compounds, we previously identified CdhR and GbdR as members of the AraC transcription factor family that regulate catabolism of the quaternary amine compounds carnitine and glycine betaine, respectively. In this study, our goal was to further characterize regulation of carnitine catabolism by the transcription factor CdhR. CdhR binds in a concentration-dependent manner upstream of the carnitine catabolism operon promoter (P_*caiXcdhCABhocS*_). We identified the CdhR binding site and determined that it overlaps with the GbdR binding site in the *caiX-cdhR* intergenic region. Carnitine catabolism is repressed by glucose and glycine betaine, and here we show this happens at the transcriptional level. Furthermore, we show that CdhR enhances its own expression and that GbdR contributes to *cdhR* expression by enhancing the level of basal expression. The intertwined regulation of *caiX* and *cdhR* transcription by GbdR and CdhR suggests that carnitine catabolism is under tight but tuneable control.

**IMPORTANCE** Pathogens must metabolize host-derived compounds during infection and properly regulate the responsible pathways. Carnitine is a common eukaryotic-associated quaternary amine compound that can be catabolized by *Pseudomonas aeruginosa*. Here we expand on our understanding of how this metabolic pathway is regulated and provide details on how carnitine catabolism is intertwined with glycine betaine catabolism at the level of transcriptional control.

## Abstract (precis)

Pathogens must metabolize host-derived compounds during infection and properly regulate the responsible pathways. Carnitine is a common eukaryotic-associated quaternary amine compound that can be catabolized by *Pseudomonas aeruginosa*. Here we expand on our understanding of how this metabolic pathway is regulated and provide details on how carnitine catabolism is intertwined with glycine betaine catabolism at the level of transcriptional control.

## INTRODUCTION

**FIG 1:**
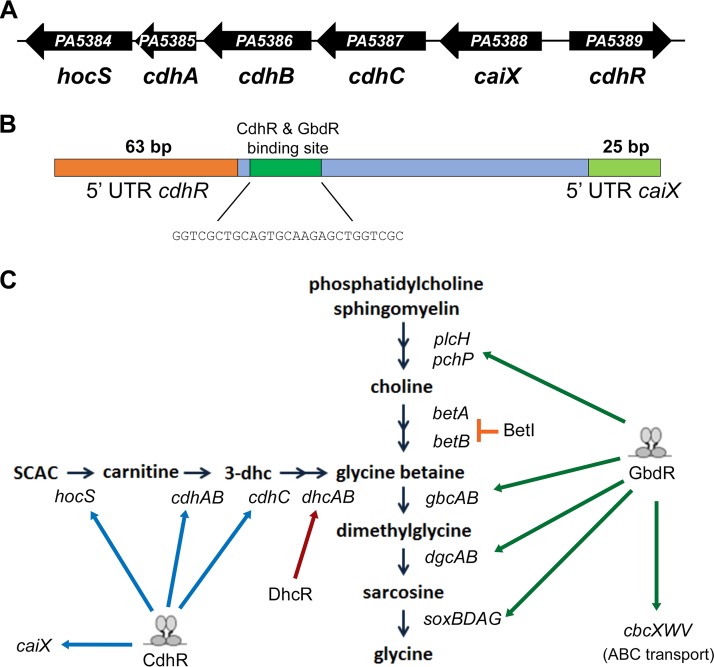
Diagram of the *P. aeruginosa* PAO1 carnitine catabolism operon and the catabolic pathway. (A) Arrows represent the individual open reading frames of the carnitine catabolism operon and the regulator *chdR*. Below the arrow is the designated gene name. (B) Diagram of the *cdhR-caiX* intragenic region organized such that *caiX* transcription occurs left to right. The orange box denotes the *cdhR* 5′ UTR, the dark green box marks the position of the CdhR and GbdR binding sites (CdhR binding sequence listed below), and the light green box denotes the *caiX* 5′ UTR. (C) Diagram of the converging carnitine and choline catabolism pathways. Black arrows represent an enzymatic step in the catabolic pathway, and the gene names are italicized below. The blue arrows represent positive regulation by either CdhR or GbdR, and the T-bar represents repression by BetI.

*Pseudomonas aeruginosa* is an opportunistic Gram-negative pathogen found in a wide variety of environments, often enriched in the drinking water distribution system, from which it can readily contaminate surfaces and medical devices in hospitals (1–4). The ability to transition from the preinfection niche to the host is likely a key to its success as a pathogen. *P. aeruginosa* encodes diverse pathways for metabolism of host-derived and host-independent carbon and nitrogen sources, allowing it to survive and thrive while undergoing these environmental transitions.

l-Carnitine (here referred to as “carnitine”) and *O-*acylcarnitines are quaternary amine compounds abundant in host tissues, one function of which is to shuttle fatty acids in and out of the mitochondria for β-oxidation in animals (5). There are no animal enzymes that can degrade carnitine (6): consequently any degradation in the host is due to bacteria, either by an aerobic pathway like *P. aeruginosa* (7) or an anaerobic pathway like many bacteria in the mammalian intestine (8). *P. aeruginosa* can acquire carnitine from the environment by import through the somewhat promiscuous ABC transporter CbcWV using the CaiX periplasmic substrate binding protein (9). The enzymes required for carnitine or short-chain acylcarnitine catabolism in *P. aeruginosa* are encoded in the carnitine catabolism operon, *caiX-cdhCAB-hocS* (Fig. 1A), enabling metabolism for osmoprotection, virulence factor induction, and nutrition (10–13). Medium- and long-chain acylcarnitines, with the exception of octanoylcarnitine, can be used as sole carbon sources as well (10), but the enzymes required for the hydrolysis of these compounds have not been identified.

Part of the metabolic flexibility of *P. aeruginosa* can be attributed to its large repertoire of regulatory proteins—more than 9% of the genome is dedicated to transcriptional regulation (14). One such regulator is CdhR, which is divergently transcribed from the carnitine catabolism operon and is required for growth on carnitine and induction of the carnitine operon (11). Aerobic carnitine degradation (Fig. 1B) leads to the formation of glycine betaine (GB), the catabolic genes for which are transcriptionally regulated by GbdR (15). GbdR and CdhR not only regulate catabolism of related quaternary amine compounds, but both are AraC family transcription factors that belong to the same glutamine amidotransferase-1-like (GATase-1) transcriptional regulator subfamily (GATRs) and are similar in sequence (62% positive and 44% identical) (16, 17).

In this study, we expand our understanding of the regulation of carnitine catabolism by identifying the CdhR binding site, determining essential binding site residues, and demonstrating that catabolite repression of carnitine catabolism by glucose and glycine betaine functions at the level of transcription of the carnitine operon. We show that GbdR can bind the intergenic region of *caiX*-*cdhR* in an orientation supporting regulation of *cdhR*. Finally, CdhR positively regulates its own expression in the presence of carnitine but represses basal expression in the absence of ligand, a repression that is alleviated when GbdR is present, suggesting a potential hierarchy of CdhR and GbdR binding at their overlapping binding sites.

## RESULTS

### Mapping the *caiX* promoter region.

The carnitine catabolism operon, *caiX-cdhCAB-hocS* (Fig. 1A), is driven from a promoter located between *caiX* and *cdhR* (Fig. 1B) and encodes proteins that are responsible for the hydrolysis of short-chain acylcarnitines (10) and degradation of carnitine to glycine betaine (GB) (11, 18) (Fig. 1C). *cdhR* is divergently transcribed from the carnitine catabolism operon (Fig. 1A) and was previously shown to be required for induction of *caiX* and growth on carnitine (11). Primer extension was used to define the transcriptional start site of *caiX* and *cdhR*. Plasmids containing the target regions were used to increase RNA copy number, particularly for *cdhR*, as native transcripts are at low abundance (19). The length of the *caiX* primer extension product placed the transcriptional start site 25 bases upstream of the translational start site at a thymine residue. The length of the *cdhR* primer extension product placed the transcriptional start site 63 bases upstream of the translational start site at a cytosine. The relative sizes of the two untranscribed regions (UTRs) and their spacing compared to the CdhR binding site are shown in Fig. 1B.

**FIG 2:**
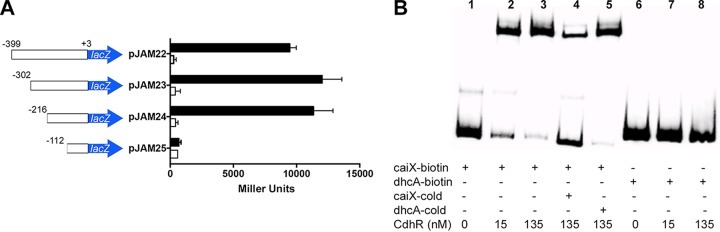
Mapping the *caiX* promoter and its CdhR binding site. (A) Transcriptional fusions of *lacZ* to the upstream region of *caiX* start at bp +3 from the *caiX* transcriptional start site and end at base pairs marked in the figure. *P. aeruginosa* PA14 strains carrying each construct were grown in MOPS with 20 mM pyruvate and 20 µg·ml^−1^ gentamicin, with (black bars) or without (white bars) 1 mM carnitine. β-Galactosidase activities for these *caiX* transcriptional fusions are reported in Miller units. Error bars represent standard deviation from three biological replicates, and results are representative of three independent experiments. (B) EMSA with biotin-labeled *caiX* promoter DNA probe alone (lane 1) or with increasing concentrations of purified MBP-CdhR (lanes 2 and 3). An unlabeled (cold) *caiX* probe was used to compete for binding of MBP-CdhR from the labeled probe (lane 4). An unrelated *dhc* DNA probe was used to show specificity of MBP-CdhR binding to *caiX* (lanes 5 to 8).

To expand upon our understanding of CdhR’s role in carnitine catabolism, we narrowed down the binding site of CdhR by promoter mapping of P_*caiX*_ using four sequentially shorter *lacZYA* transcriptional reporters. The constructs end at bp +3 from the *caiX* transcriptional start site and begin at bp −399 (pJAM22), bp −302 (pJAM23), bp −216 (pJAM24), and bp −112 (pJAM25) (Fig. 2A). In the presence of carnitine, all constructs except pJAM25 were induced, indicating that the binding site of CdhR is between bp −216 and −112 or overlaps −112 (Fig. 2A) in relation to the transcriptional start site.

Based upon CdhR-dependent transcriptional induction of *caiX* (11) (Fig. 2A), we assayed the capability of purified maltose binding protein (MBP)-CdhR to bind to the upstream activation sequence (UAS) of *caiX*. Using biotin-labeled *caiX* UAS as the DNA probe and purified MBP-CdhR, electrophoretic mobility shift assays (EMSAs) revealed that MBP-CdhR binds the *caiX* UAS in a concentration-dependent manner (Fig. 2B, lanes 1 to 3). The binding interaction between MBP-CdhR and *caiX* probe can be competed with unlabeled *caiX* probe (lane 4), but not an unlabeled *dhcA* probe (lane 5), and MBP-CdhR does not shift the nonspecific UAS, *dhcA* (lanes 6 to 8). These data demonstrate that MBP-CdhR binds specifically to the *caiX* UAS.

### Identification of the CdhR binding site sequence and bases required for induction of *caiX.*

**FIG 3:**
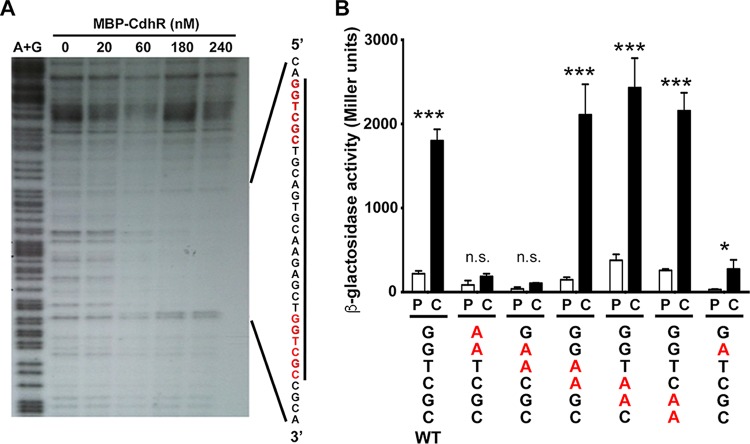
The CdhR binding site and key residues for CdhR-dependent induction. (A) A DNase I footprinting assay was performed by taking the *caiX* UAS end labeled with ^32^P and adding increasing concentrations of MBP-CdhR, followed by DNase I treatment and nondenaturing 5% polyacrylamide TBE gel. The first lane of the gel is the A+G sequencing ladder, and the nanomolar concentration of MBP-CdhR is marked. (B) The *caiX* enhancer site was mutated by changing two bases at a time (in red and underlined) in the *caiX* distal binding site to adenosines and fused to *lacZ*. The *P. aeruginosa* PA14 wild type carrying each of the plasmids was grown in MOPS with 20 mM pyruvate at 20 µg·ml^−1^, with or without 1 mM carnitine for 4 h, and then β-galactosidase activity was reported as Miller units. Error bars represent standard deviations from three biological replicates, and results are representative of three independent experiments. Data were analyzed using a two-way analysis of variance (ANOVA) with a Sidak’s multiple-comparison posttest comparing each mutant’s pyruvate to carnitine. Abbreviations: P, pyruvate; C, carnitine; n.s., not significant; *, *P* < 0.05; ***, *P* < 0.001.

Promoter mapping and EMSAs determined the region of DNA necessary for carnitine-dependent *caiX* transcriptional induction and CdhR binding (Fig. 2). To further characterize the CdhR DNA contact site within the *caiX* UAS, we employed DNase I footprinting. There was a characteristic and site-specific protection of *caiX* UAS DNA as the MBP-CdhR concentration increased (Fig. 3A). Comparing the zone of protection to the A+G ladder, we were able to identify the CdhR contact region, stretching 34 bp (Fig. 3A). The CdhR binding site reveals that each half-site contains the sequence GGTCGC with a 15-bp spacer, which is very similar to the binding sites of three other related *P. aeruginosa* transcription factors, GbdR (17), SouR (20), and ArgR (21). To determine the importance of the half-site residues, we made mutations in the distal half-site by systematically changing partially overlapping dinucleotides to adenines (pJAM122 to pJAM127). Induction from these mutated *cdhR* binding sites demonstrated that the second guanine residue in the half-site is important for *caiX* expression, but complete abolishment of carnitine-dependent induction requires an additional mutation of the adjacent thymine residue (Fig. 3B).

### Glucose and glycine betaine repress the transcription of *caiX.*

**FIG 4:**
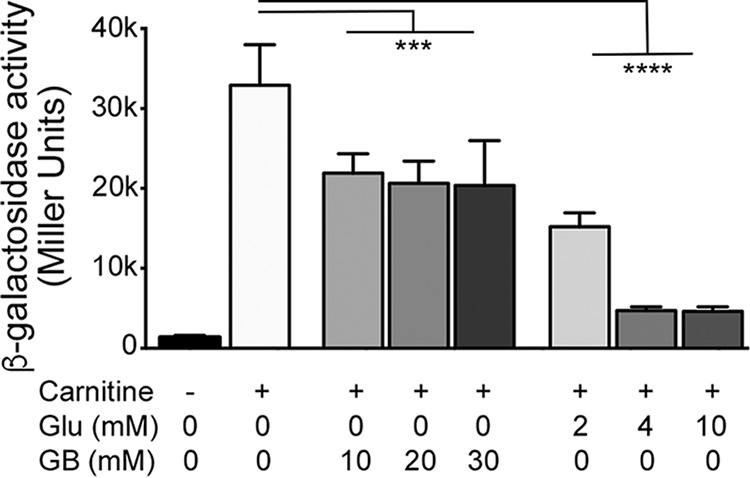
*caiX* transcription is repressed by glucose and glycine betaine. *P. aeruginosa* PA14 with P*_caiX_-lacZ* (pJAM22) was grown in MOPS medium with 20 mM pyruvate and 20 µg·ml^−1^ gentamicin, with or without 1 mM carnitine. Glucose or glycine betaine was added at the millimolar concentrations noted. Cultures were induced for 4 h prior to measurement of β-galactosidase activity. Error bars represent standard deviations from three biological replicates, and results are representative of three independent experiments. Data were analyzed using a one-way ANOVA with a Dunnett’s multiple-comparison posttest comparing each condition to the condition with carnitine alone. Bars denote that all data underneath are different from carnitine alone with the same statistical certainty. Abbreviations: Glu, glucose; GB, glycine betaine; ***, *P* < 0.001; ****, *P* < 0.0001.

In *P. aeruginosa*, the enzymatic activity of carnitine dehydrogenase (CDH) declines until no longer detectable if the cells are switched from carnitine to glucose as the sole carbon source (22). CDH activity oscillates when carnitine is the sole carbon source, predicted to occur when the catabolic product glycine betaine is produced, resulting in initiation of a negative-feedback oscillation loop (22). We used the *caiX-lacZYA* transcriptional reporter (pJAM22) to determine if catabolite repression is transcriptionally regulated. Maximal catabolite repression is seen at 4 mM glucose, while GB represses transcription to a lesser extent and at a higher concentration (Fig. 4).

### CdhR can bind, but does not regulate, the ABC transporter *cbcXWV.*

**FIG 5:**
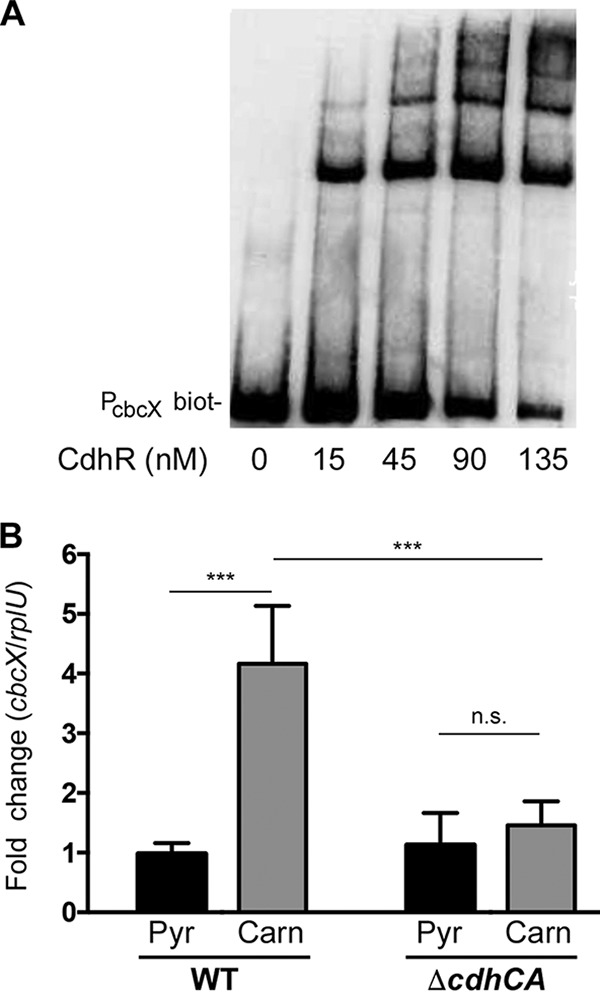
CdhR binds but does not regulate *cbcXWV* expression. (A) EMSA with a biotin-labeled (biot-) *cbcX* upstream region and purified MBP-CdhR in increasing concentrations. (B) Relative expression of *cbcX* was calculated based on the expression in WT pyruvate normalized to the *rplU* transcript using qRT-PCR. Three biological samples were run in triplicate, and the graph represents the mean values and standard deviation. Data were analyzed using a two-way ANOVA with a Tukey’s multiple comparison posttest comparing all strains and conditions to each other. Ends of the bars denote the comparison groups shown. Abbreviations: Pyr, pyruvate; Carn, carnitine; n.s., not signiﬁcant; ***, *P* < 0.001.

After establishing the CdhR binding sequence, GGTCGC-[N15]-GGTCGC, we searched for this sequence in the *P. aeruginosa* PAO1 genome using the DNA motif search tool from the *Pseudomonas* Genome Database website (23). Only two identical sites were identified within intergenic regions: *caiX-cdhR* and *cbcX-sdaB*, both of which are involved in carnitine metabolism (9, 11). CbcXWV is an ABC transporter, and the core transporter proteins CbcWV are required for growth on carnitine along with the substrate binding component CaiX (9). An MBP-CdhR EMSA with the *cbcXWV* UAS probe showed MBP-CdhR binding in a concentration-dependent manner (Fig. 5A), but quantitative reverse transcription-PCR (qRT-PCR) revealed that carnitine cannot support induction of *cbcX* (Fig. 5B). Deletion of *cdhCA* eliminates production of GB from carnitine (Fig. 1B) (11); therefore, for carnitine to lead to *cbcXWV* induction, GB must be produced to enable *cbcXWV* induction via GbdR (17).

### Roles of GbdR and CdhR at the *caiX-cdhR* intergenic region.

**FIG 6:**
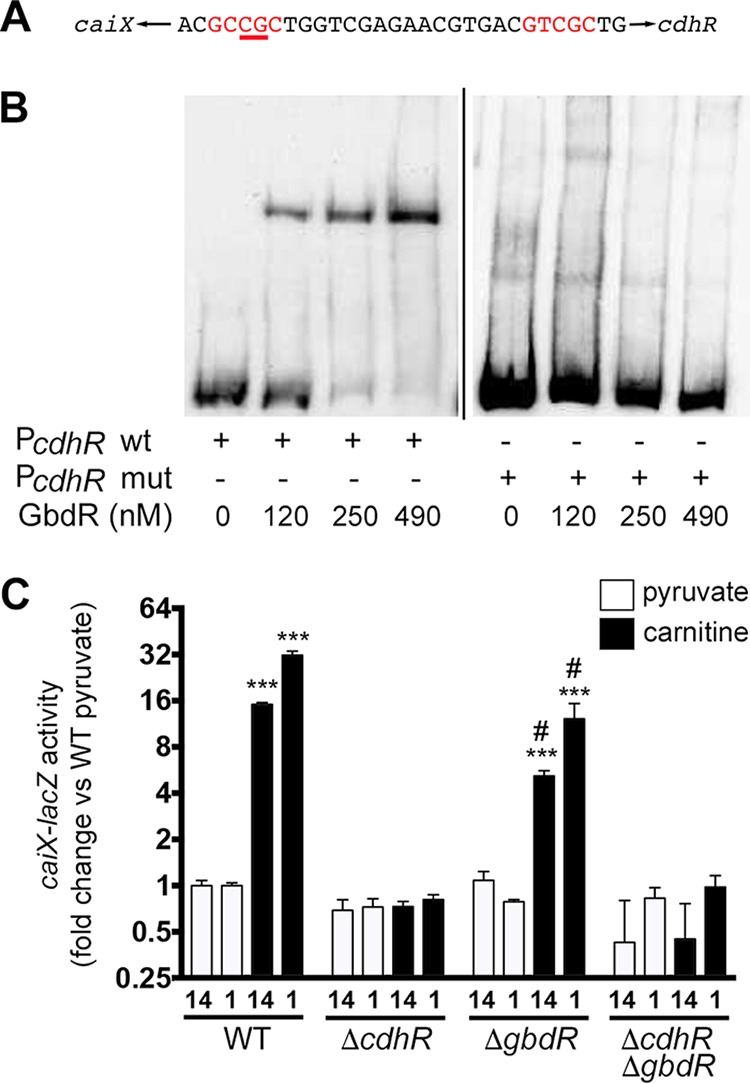
GbdR binds the *caiX-cdhR* intergenic region but does not induce transcription of *caiX*. (A) EMSAs were performed with increasing concentrations of purified MBP-GbdR with either the biotin-labeled *cdhR* wild-type probe or a mutant binding site probe. The mutated probe has the distal half-site CG residues (in relation to *cdhR*) changed to AA. (B) β-Galactosidase assay with a *caiX-lacZ* reporter plasmid (pJAM22) in wild-type, Δ*cdhR*, Δ*gbdR*, or Δ*cdhR* Δ*gbdR* strains of both PA14 (14) and PAO1 (1) grown in MOPS, 20 mM pyruvate, and 20 µg·ml^−1^ gentamicin. Induced cultures have an additional 1 mM carnitine. Error bars represent standard deviations from three biological replicates, and results are representative of three independent experiments. Data were analyzed with two-way ANOVA with a Tukey’s multiple-comparison test comparing all mutants and conditions within a given strain. (PA14 was not compared to PAO1.) Abbreviations: ***, *P* < 0.001 compared to WT with pyruvate; #, *P* < 0.01 compared to WT with carnitine.

The ability of CdhR to bind a known member of the GbdR regulon (*cbcX*) (17, 24), the detection of the GbdR binding consensus in the *caiX-cdhR* intergenic region (17), and the overlapping positions of the CdhR and GbdR consensus sites in the *caiX* UAS led us to investigate the role of GbdR in carnitine regulation. We predicted that GbdR would be able to bind the *cdhR* UAS, and an EMSA with purified MBP-GbdR demonstrated that MBP-GbdR binds the *caiX*-*cdhR* intergenic probe in a concentration-dependent manner (Fig. 6A). When the conserved CG residues for the GbdR-binding distal half-site were mutated to AA (GCCGC to GCAAC), binding was lost (Fig. 6A), as previously seen for similar mutations in the *plcH* and *choE* distal half-sites (17). Since GbdR binds the *caiX-cdhR* intergenic region, induction of *caiX* (pJAM22) was assessed in the wild type (WT) and a *gbdR* deletion mutant in both PA14 and PAO1 backgrounds. The *gbdR* deletion mutant has less induction of P*caiX-lacZ* (Fig. 6C), but this defect is likely due to a defect in carnitine import (9) (Fig. 4).

**FIG 7:**
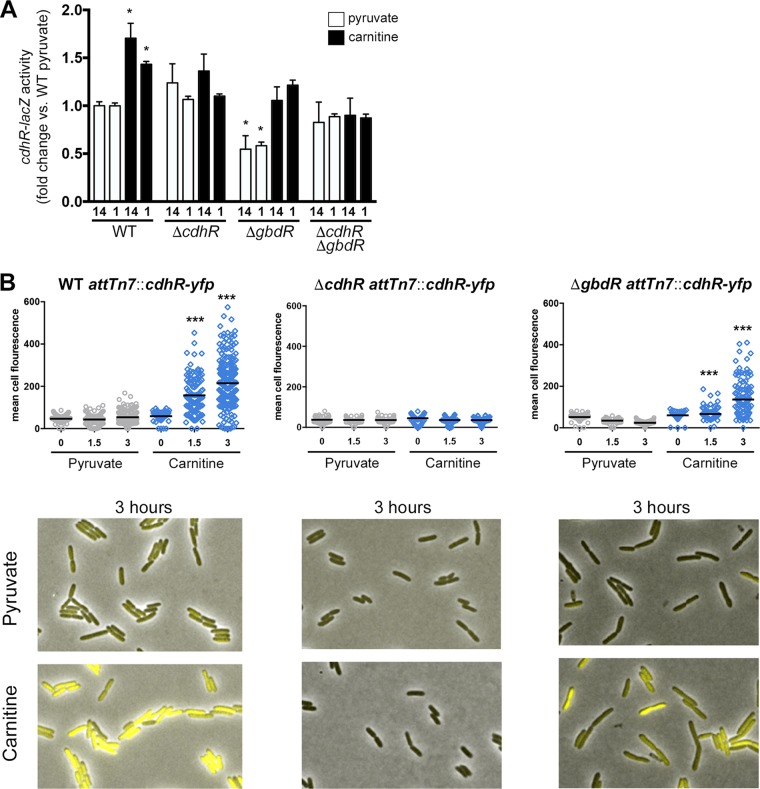
CdhR promotes *cdhR* expression, and GbdR dampens basal repression. (A) WT, Δ*cdhR* Δ*gbdR*, and Δ*cdhR* Δ*gbdR* strains in both PA14 (14) and PAO1 (1) backgrounds carrying a *cdhR-lacZ* translational plasmid reporter (pJAM135) were grown in MOPS with 20 mM pyruvate and 20 µg·ml^−1^ gentamicin, with or without 1 mM carnitine, and β-galactosidase activity was reported as fold change over WT pyruvate. Data were analyzed using a two-way ANOVA with a Sidak’s multiple-comparison posttest comparing to the WT pyruvate condition within each strain. (PA14 was not compared to PAO1.) *, *P* < 0.05. (B) PAO1 WT, Δ*cdhR*, and Δ*gbdR* strains, all with the translational fusion *cdhR-yfp* integrated at the *att*Tn7 site, were grown on MOPS agar pads with 20 mM pyruvate and with or without 1 mM carnitine. Cells were imaged under phase-contrast and YFP fluorescence every 10 min at 32°C. Data were analyzed using a one-way ANOVA with a Dunnett’s multiple-comparison posttest comparing each time point within a strain to pyruvate at time zero (*t* = 0). ***, *P* < 0.001.

**FIG 8:**
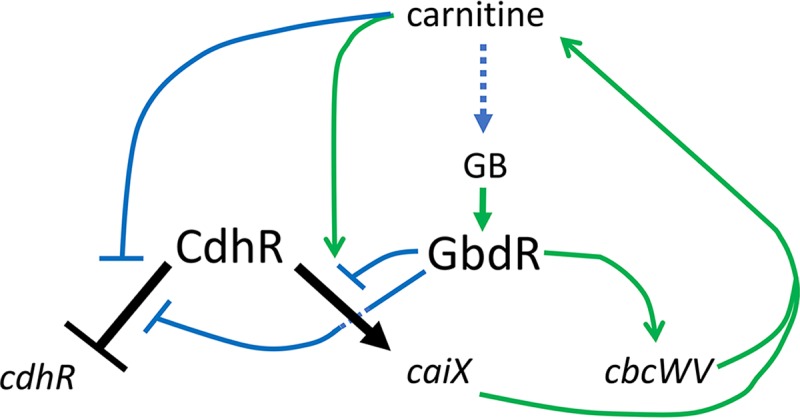
Regulation of *caiX* and *cdhR* by CdhR and GbdR. Shown is a genetic network diagram of the interactions known and proposed in this study. Arrows indicate positive interactions (induction or activation), and T-bars indicate negative interactions (repression or other inhibition). The regulatory steps are black for CdhR and green or blue for interactions between other members in the network. The dotted blue line from carnitine to glycine betaine (GB) marks the metabolic conversion noted in Fig. 1C.

After establishing that CdhR and GbdR bind the intergenic region of *caiX-cdhR* (Fig. 2B, 3A, and 6), we wanted to determine how these two transcription factors (TFs) impacted *cdhR* expression. Using two different translational reporter fusions—one carried on a plasmid in both PA14 and PAO1 backgrounds and one integrated into the chromosome at the *att*Tn*7* site—it became apparent that CdhR has a role in its own expression. In the wild type, carnitine increased expression of *cdhR* compared to the basal expression level (pyruvate) (Fig. 7A). In the absence of *gbdR*, carnitine still induces *cdhR*, but basal expression of *cdhR* is decreased compared to that of the wild type and a *cdhR* deletion mutant, suggesting that GbdR functions to relieve repression at this locus (Fig. 7A). The similarity of the activity in the double deletion mutant with the *gbdR* single deletion in the presence of carnitine suggests that carnitine detection by CdhR allows relief of the baseline CdhR-dependent repression. The effects of CdhR and GbdR on *cdhR* expression are also seen with single-cell expression levels (Fig. 7B), showing both the general effects seen with the population assessment (Fig. 7A), as well as the heterogeneity in individual cell expression. These findings are summarized in a genetic model (Fig. 8).

## DISCUSSION

The metabolic diversity of *P. aeruginosa* is controlled by a vast set of regulators, including one-component transcription factors that are often critical for regulating catabolism of alternate nutrient sources (25). One such transcription factor family that can control carbon metabolism and virulence is the AraC family (26, 27). In this study, we expand on our understanding of carnitine catabolism and show how two AraC family transcription factors, CdhR and GbdR, whose metabolic pathways converge at glycine betaine (GB) (Fig. 1C), are intertwined (Fig. 8). We identified the CdhR binding site in the *caiX-cdhR* intergenic region and through reporter and DNA binding assays reveal this site as an additional GbdR binding site. Through reporter fusions, we were able to show that CdhR regulates its own expression and is responsive to carnitine. We also show that GbdR binding to the *caiX-cdhR* region assists in regulating carnitine catabolism by inhibiting repression of *cdhR* transcription, thus maintaining the basal *cdhR* expression level.

The first question we addressed was how CdhR binds and regulates the carnitine operon. CaiF in *Escherichia coli* is the only other carnitine regulator that has been characterized to date, and it is a degenerate AraC TF that lacks the traditional N-terminal domain but maintains the helix-turn-helix (HTH) DNA binding domains (28). The CaiF amino acid sequence is 13% identical and 23% similar with 44% gaps compared to CdhR, with most of the similar regions within the HTH domains, therefore likely functioning in a manner much different than CdhR. *caiF* is transcribed in the opposite direction from the *E. coli* carnitine metabolism operon (*caiTABCDE*), which codes for utilization of carnitine as a terminal electron acceptor generating γ-butyrobetaine and binds to inverted repeats in the *caiF* UAS (28, 29). *P. aeruginosa* and other Gram-negative bacteria, capable of utilizing carnitine as a carbon and/or nitrogen source, contain a carnitine catabolism operon capable of generating glycine betaine and are organized in similar fashion to one another with an AraC family transcription factor divergently transcribed from the catabolic operon (Fig. 1A) (30). CdhR binds to direct repeats, and the binding site is upstream of the *caiX* promoter (Fig. 1B and 3A), categorizing CdhR as a class I activator (binds upstream and recruits RNA polymerase via the C-terminal domain of the alpha subunit [31]) that requires both half-sites for induction of *caiX* (Fig. 3B).

Kleber and Aurich analyzed the activity of carnitine dehydrogenase (CDH) with respect to glucose and glycine betaine (GB) and showed that glucose, as a preferred carbon source, is catabolite repressive (22), whereas glycine betaine leads to repression of CDH activity, resulting in oscillations of activity as carnitine is catabolized to glycine betaine (22). Our data demonstrate that repression of carnitine catabolism by glucose and glycine betaine can be controlled at the level of transcription (Fig. 4). Negative feedback by glycine betaine is likely GbdR dependent based on GbdR’s capability to bind the *caiX-cdhR* intergenic region (Fig. 6B) and GbdR’s responsiveness to GB (32). *P. aeruginosa* maintains intracellular glycine betaine pools, and GbdR fine-tuning of carnitine catabolism may be directly related to sustaining the homeostatic levels of glycine betaine, as the GB pool has a physiological impact on nutrients, osmoprotection, and virulence (33).

We performed an alignment using the Pseudomonas.com DNA motif search tool of the newly identified CdhR binding sequence to the PAO1 genome and identified two intergenic regions: *caiX-cdhR* and *cbcX-sdaB* (23). This led us to investigate if CdhR contributes to the regulation of carnitine import by the ABC transporter CbcWV in association with CaiX, which is required for growth on carnitine (9). Even though CdhR binds to the *cbcXWV* UAS *in vitro*, it does not contribute to *cbcXWV* expression (Fig. 5). We propose that an unknown transporter imports carnitine, which is metabolized to GB, and this GB drives expression of *cbcXWV* in a GbdR-dependent manner, which coupled with CdhR-dependent expression of *caiX* allows for a larger flux of carnitine needed to support growth (9). This is similar to the mechanism of choline import for *cbcXWV* induction, which is termed priming (24).

We previously characterized the GbdR regulon and identified the intergenic region of *caiX-cdhR* to have a GbdR binding site (17). Here we report that GbdR binds the *caiX-cdhR* intergenic region *in vitro* and the conserved CG residues are necessary for GbdR binding, as mutation of these residues to AA results in loss of binding (Fig. 6A). Based upon the conserved residues being located in the GbdR distal half-site (17), the orientation of GbdR binding is likely toward *cdhR* activation and not *caiX*. Interestingly, the GbdR and CdhR binding sites overlap in the *caiX-cdhR* intergenic region, which led to the hypothesis that GbdR has a role in regulating carnitine catabolism and, in particular, *cdhR*.

We propose a model (Fig. 8) showing the genetic network containing CdhR and GbdR. In the absence of carnitine, CdhR binds to its *caiX-cdhR* intergenic target sequence site in a manner that inhibits *cdhR* expression. GbdR competes for this binding site and limits CdhR-dependent inhibition of *cdhR*. Analysis of the DNA sequence up- and downstream of the CdhR binding site reveals multiple CdhR half-sites. These half-sites could participate in inhibition of CdhR basal expression by looping. CdhR inhibition of its own expression may be similar to the AraC “light-switch” mechanism, in which the regulator binds upstream sites to loop DNA and restrict polymerase access to the promoter (34). Another possibility is that CdhR oligomerizes along the DNA, nucleated at these half-sites, to dampen *cdhR* basal expression. In this model, GbdR would compete for binding, relieving CdhR-dependent *cdhR* repression. Upon CdhR detection of carnitine, a change occurs allowing increased expression of *cdhR* and *caiX* (Fig. 7). As the catabolic product GB builds up, the cell controls the flux of carnitine catabolism by GB-dependent transcriptional repression (Fig. 4), which is likely regulated by GbdR (Fig. 8).

In conclusion, we show that CdhR regulates the carnitine catabolic operon by directly binding the *caiX* UAS, and this is likely the only site in the genome where its potential binding is effective in altering gene transcription. We also show that catabolic repression of carnitine catabolism can function through direct repression of carnitine catabolic operon transcription. Finally, we show the role of GbdR in regulation at this site. These data suggest a system for fine-tuning carnitine catabolism in relation to other carbon sources and that both GbdR and CdhR alter transcription from the *caiX-cdhR* intergenic regulatory region.

## MATERIALS AND METHODS

### Strains and growth conditions.

*P. aeruginosa* wild-type strains PAO1 and PA14 and their derivatives (Table 1) were maintained on *Pseudomonas* isolation agar (PIA [Difco]) plates or Lennox broth (LB) liquid, and when necessary 50 or 40 µg·ml^−1^ gentamicin was added to the media, respectively. *Escherichia coli* NEB5α or T7 Express *E. coli* (NEB C3016) cells were maintained on LB plates with 10 µg·ml^−1^ gentamicin, LB liquid with 7 µg·ml^−1^ gentamicin, LB plates or liquid with 125 µg·ml^−1^ carbenicillin, or LB plates or liquid with 100 µg·ml^−1^ kanamycin.

**TABLE 1:** Strains and plasmids used in this study

Strain or plasmid	Genotype or description	Reference or source
Strains		
* P. aeruginosa* PAO1		
MJ79	Wild type	14
MJ80	Δ*gbdR*	15
JM236	Δ*cdhR*	This study
JM253	Wild type *att*Tn*7*::88-89intYFPCFP-2	This study
JM339	Δ*cdhR att*Tn*7*::88-89intYFPCFP-2	This study
JM340	Δ*gbdR att*Tn*7*::88-89intYFPCFP-2	This study
MJ784	Δ*gbdR* Δ*cdhR*	This study
* P. aeruginosa* PA14		
MJ101	Wild type	42
MJ11	Δ*cdhR*	11
MJ26	Δ*gbdR*	15
MJ262	Δ*cdhCA*	11
JM179	Δ*cdhR* Δ*gbdR*	This study
* E. coli*		
MJ340	Wild-type S17λpir	
DH5α	NEB C2987	NEB
T7Express	NEB C3016	NEB

Plasmids		
pMQ30	Suicide vector, Gm^r^	36
pMQ80	High-copy-no. *Pseudomonas* vector, Gm^r^	36
pMal-C2X	T7-expressing vector, MBP N-terminal tag, Amp^r^	NEB
pTNS2	Plasmid carrying *att*Tn*7* transposase	43
pUC18-mini-Tn*7*T-Gm	Gm^r^ on mini-Tn*7*T	35
pUC18-mini-Tn*7*T-Gm-*eyfp*	Gm^r^ on mini-Tn*7*T with YFP	35
pUCP22	High-copy-no. *Pseudomonas* stabilization vector, Gm^r^	44
pMW5	*lacZYA* in pUCP22	32
pMW79	PA14 genomic clone of *PA5380-PA5389* in pMQ80	11
pPA5380KO	*gbdR* deletion construct in pEX18-Gm	15
pJAM22	Promoter *caiX-lacZYA* transcriptional fusion A	This study
pJAM23	Promoter *caiX-lacZYA* transcriptional fusion B	This study
pJAM24	Promoter *caiX-lacZYA* transcriptional fusion C	This study
pJAM25	Promoter *caiX-lacZYA* transcriptional fusion D	This study
pJAM50	*PA5389* in pMal-C2X	This study
pJAM76	YFP-CFP in pMQ80	This study
pJAM86	CFP *PA5388-PA5389* intergenic region YFP in pUC18mini, DR2	This study
pJAM90	*PA5389* deletion construct in pMQ30	This study
pJAM122	Promoter *caiX-lacZYA* transcriptional fusion	This study
pJAM123	Promoter *caiX-lacZYA* mut 1 transcriptional fusion	This study
pJAM124	Promoter *caiX-lacZYA* mut 2 transcriptional fusion	This study
pJAM125	Promoter *caiX-lacZYA* mut 3 transcriptional fusion	This study
pJAM126	Promoter *caiX-lacZYA* mut 4 transcriptional fusion	This study
pJAM127	Promoter *caiX-lacZYA* mut 5 transcriptional fusion	This study
pJAM130	Promoter *caiX-lacZYA* mut 6 transcriptional fusion	This study
pJAM131	C terminus of *lacZ* in pUCP22	This study
pJAM135	P_*cdhRlacZYA*_ in pUCP22	This study

### Deletion constructs.

A deletion of *PA5389* (*cdhR*) in PAO1 was made in the wild-type background (MJ79). The upstream and downstream regions of *PA5389* were PCR amplified from PAO1 genomic DNA with the primers 5389GOIF1KpnI, 5389SOEGOIR1, 5389SOEGOIF1, and 5389GOIR1BamHI (Table 2). The splice overlap extension PCR product was ligated into the Zero Blunt plasmid pCR-Blunt (Invitrogen), excised with EcoRI, and ligated into similarly cut pMQ30 to generate pJAM90. Conjugation of donor *E. coli* S17λpir carrying pJAM90 with PAO1 and subsequent screening were done as previously described (10), generating strain JM236. The deletion strain, JM179, was verified by lack of growth on carnitine and by PCR.

**TABLE 2:** Primers used in this study

Primer	Sequence (5′ to 3′)^*a*^
Deletion constructs	
5389GOIF1KpnI	ATAGGGTACCGAAGAACACCACCCACTGCT
5389SOEGOIR1	AAGTACGAAGGCGACTCGACCATGGAGAAGCCCATTACCGAGAAGC
5389SOEGOIF1	GCTTCTCGGTAATGGGCTTCTCCATGGTCGAGTCGCCTTCGTACTT
5389GOIR1BamHI	ATCGTCTTCGCTGTTTTTCC
Protein expression construct	
5389Mal-c2xF	GCATCAGAATTCTCCCAGGACTTCTGGTTTCT
5389Mal-c2xR	GCATCAAAGCTTTCAGCCTCGCTCAGCTCGA
Primer extension	
5388primerextension	5′-Fluorescein 6-FAM-ACTGGCCAGGATCAGCAGG
5389primerextension	5′-Fluorescein 6-FAM-AGACAGTATCGGCCTCAGGAA
EMSA probes	
PA5388promF3	AAGCTTGTGCCAGCGGTAGAGGTC
PA5388promR	TGAGGTACCTTGATTGTTTTTCTGCGAGGT
PA5388promRbiot	Biotin-TTGATTGTTTTTCTGCGAGGT
5389EMSA-F	ATGAAAGCTTGCAGCAGGAGAAACCAGAAG
5389EMSA-R-biot	Biotin-TTGATTGTTTTTCTGCGAGGT
5389EMSA-Mut3F	GGACGGCGGCGAAGCGCACTGCGAAGACC
cbcXprom-F	CCGGCAAAGACCACTATGAT
cbcXprom-R-biot	Biotin-GAACTCCTCTGCAGGGTAAGG
dhcprom-F-biot	Biotin-GAGGCTTTCCTCCAGGCTCT
dhcprom-R	GGATGGTACCCTCTTCCGGCTCTTGTGATT
dhcprom-F	GAACTCCTCTGCAGGGTAAGG
Transcriptional reporters	
PA5388promR	TGAGGTACCTTGATTGTTTTTCTGCGAGGT
PA5388promF1	ATGAAAGCTTACAGCAGGTCGCCTTTCTT
PA5388promF3	AAGCTTGTGCCAGCGGTAGAGGTC
PA5388promF2	ATGAAAGCTTGCAGCAGGAGAAACCAGAAG
PA5388promF4	AAGCTTCTGCAGTGCAAGAGCTGGT
P5388pos	ATGAAAGCTTCGCTTGGCAATGGCCAGGTCGCT
P5388mut1	ATGAAAGCTTCGCTTGGCAATGGCCAAATCGCT
P5388mut2	ATGAAAGCTTCGCTTGGCAATGGCCAGAACGCT
P5388mut3	ATGAAAGCTTCGCTTGGCAATGGCCAGGAAGCT
P5388mut4	ATGAAAGCTTCGCTTGGCAATGGCCAGGTAACT
P5388mut5	ATGAAAGCTTCGCTTGGCAATGGCCAGGTCAAT
P5388mut6	ATGAAAGCTTCGCTTGGCAATGGCCAGATCGCT
Translational reporters	
2-lacZCtermFhindcla	GCAAGCTTATTATCGATGAGCGTGGTGGTTATGC
2-lacZCtermRsmakpn	CGGTACCCGGGGATCCTTATTTTTGACACCAGACC
YFP R HindIII	GCATCAAAGCTTATTACTTGTACAGCTCGTCCA
YFP F Kpn Sal	GACAGCGGTACCAATCGTCGACCATATGCTGAGCAAGGGCGAGG
88-89YC-DR#2ycF	GGGCACCACCCCGGTGAACAGCTCCTCGCCCTTGCTCAGCATGGGGCGCTCCGGGGTTGA
88-89YC-DR#2ycR	CGGCACCACGCCGGTGAACAGCTCCTCGCCCTTGCTCAGCATCGGTCTCCCCTCGTGCGG

^a^ 6-FAM, 6-carboxyfluorescein.

A double mutant of *cdhR* and *gbdR* in PA14 was made using the PA14 Δ*gbdR* strain MJ26 as the recipient strain for mating with *E. coli* S17λpir carrying pJAM90 as described above. The double mutant strain was verified by PCR. The PAO1 *cdhR gbdR* double mutant was made using the PAO1 Δ*cdhR* strain JM236 as the recipient strain for mating with *E. coli* S17λpir carrying pPA5280KO (15). The double mutant was verified by PCR and its inability to grow on glycine betaine.

### Construction of transcriptional reporter constructs.

Promoter mapping of *PA5388* (*caiX*) was done using four different truncations of the *caiX* upstream region cloned as transcriptional reporter constructs. pMW79 was used as the PCR template for amplification of the upstream region of *caiX*, using PA5388promR as the reverse primer for all products with the forward primers PA5388promF1, PA5388promF2, PA5388promF3, and PA5388promF4. These four PCR products were ligated into the pCR-Blunt, digested with KpnI and HindIII, and ligated into similarly cut pMW5 to yield four different P*5388lacZYA* transcriptional fusions (pJAM22 to pJAM25). Each was transformed by electroporation into PA14 wild type and selected on PIA-gentamicin plates.

To assess which residues were essential for induction of *caiX*, seven different transcriptional reporters were constructed by amplifying the *caiX* binding site from pMW79 using the PA5388promR primer and seven different forward primers: P5388pos, P5388mut1, P5388mut2, P5388mut3, P5388mut4, P5388mut5, and P5388mut6. PCR products were digested with HindIII and KpnI and ligated into similarly cut pMW5. The resulting plasmids, pJAM122 to pJAM127 and pJAM130, were transformed into PA14 wild type by electroporation and selected on PIA-gentamicin.

### Construction of *cdhR* translational reporter constructs.

To determine control of *cdhR*, a divergent fluorescent translational reporter with yellow fluorescent protein (YFP) and cyan fluorescent protein (CFP) on either side of the intergenic region of *PA5388* and *PA5389* was constructed. YFP was amplified using primers YFP F Kpn Sal and YFP R HindIII from the template pUC18miniTn7T-Gm-*eyfp* (35). The YFP fragment was ligated into pCR Zero Blunt plasmid, digested with HindIII and KpnI, and subsequently ligated into similarly cut pMQ80. The resulting plasmid was digested with EcoRI and SacI and ligated with an EcoRI- and SacI-cut *P. aeruginosa-*codon-biased CFP, to generate pJAM76. The *PA5388-PA5389* intergenic region was amplified using primers 88-89YC-DR#2ycF and 88-89YCDR#2ycR. pJAM76 was digested with SalI to linearize the plasmid for recombination of the *PA5388-PA5389* intergenic regions using yeast cloning in *Saccharomyces cerevisiae* via the method of Shanks et al. (36). The resultant plasmid was digested with HindIII, and the insert was ligated into similarly cut pUC18-mini-Tn7T-Gm, yielding pJAM86, which was coelectroporated with pTNS2 to insert CFP-PA5388-PA5389-YFP onto the chromosome at the *att*Tn7 site (35) of the PAO1 wild-type, PAO1 Δ*cdhR*, and PAO1 Δ*gbdR* strains.

A *PA5389* translational *lacZ* fusion was made by inserting a gBlock (IDT) that has 320 bp of the upstream region of *PA5389* into the translational start site of *PA5389* and then from the translational start site of *lacZ* to +850 bases in the *lacZ* gene into pJAM131. pJAM131 was built by PCR amplification of the C-terminal end of* lacZ* with primers 2-lacZCtermFhindcla and 2-lacZCtermRsmakpn, digested with HindIII and KpnI, and ligated into a similarly cut pUCP22. The gBlock was digested with HindIII and ClaI and ligated into similarly cut pJAM131 to make pJAM135.

### Cloning and expression of MBP-CdhR.

The plasmid pJAM50, expressing a maltose binding protein (MBP) fusion to the amino terminus of CdhR, was made by amplifying *cdhR* with primers 5389Mal-C2XF and 5389Mal-C2XR and ligating the product into pCR-Blunt. The *cdhR* coding segment was excised with EcoRI and HindIII and ligated into the similarly cut pMal-C2X (NEB), transformed by electroporation into T7 Express *E. coli* (NEB C2566), and selected on LB-carbenicillin to generate strain JM153.

To express MBP-CdhR, 1 liter of JM153 was grown at 37°C in LB with 75 µg·ml^−1^ carbenicillin to an optical density at 600 nm (OD_600_) of 0.4. The culture was induced with 1 mM isopropyl-β-d-thiogalactopyranoside (IPTG) and grown for an additional 3 h. Cells were collected by centrifugation and resuspended in 20 mM Tris HCl–150 mM NaCl (pH 7.4) with 1× Halt protease inhibitor cocktail (Thermo) and lysed by French press. DNase I was added to the lysate and incubate room temperature for 15 min. Samples were centrifuged at 13,000 rpm for 30 min at 4°C to separate the soluble and insoluble fractions. The soluble fraction was filtered through a 0.22-µm-pore filter and applied to an Affi-Gel heparin gel (Bio Rad) column. The column was washed with column buffer (10 mM KH_2_PO_4_, 150 mM NaCl [pH 7.4] in 1× phosphate-buffered saline [PBS]), and MBP-CdhR was eluted from the column with column buffer plus 1.5 M NaCl. Fractions containing MBP-CdhR were run on a 12% SDS denaturing gel and stained with Coomassie brilliant blue (Thermo) to verify purity and determine which fractions to use. Elutions containing MBP-CdhR were dialyzed in a 20,000 molecular weight cutoff (MWCO) Slidealyzer (Thermo) overnight at 4°C in buffer (20 mM Tris-HCl, 150 mM NaCl, 1 mM EDTA [pH 7.4]).

### Primer extension.

The transcriptional start sites of *caiX* and *cdhR* were determined by growing PA14 the wild type carrying pMW79 or PA14 wild type carrying pJAM135, respectively, in morpholinepropanesulfonic acid (MOPS) medium with 20 mM pyruvate, 5 mM glucose, and 20 µg·ml^−1^ gentamicin overnight at 37°C. Cells were washed, resuspended to an OD_600_ of 0.3 in MOPS supplemented with 20 mM l-carnitine or pyruvate and 20 µg·ml^−1^ gentamicin in a 12-well plate, and grown for 7 h at 37°C while shaking. Cells were collected by centrifugation, and RNA was purified using the RNeasy minikit (Qiagen) as per the manufacturer’s instructions. Primer extension was completed using the purified RNA as the template for cDNA generation using Superscript II reverse transcriptase (Invitrogen) and the 5′-fluorescein-labeled primers 5388primerextension and 5389primerextension. The cDNA products were analyzed by capillary electrophoresis to determine the size of the DNA fragment of the induced carnitine sample versus the pyruvate control sample by comparison to DNA size standards.

### β-Galactosidase assays.

Strains were grown overnight at 37°C in MOPS minimal medium supplemented with 20 mM pyruvate, 5 mM glucose, and 20 µg·ml^−1^ gentamicin. Cells were collected by centrifugation, washed, resuspended in MOPS, and inoculated into MOPS with 20 mM pyruvate and 20 µg·ml^−1^ gentamicin, with or without 1 mM carnitine as the inducing compound. Induction was carried out for 4 h at 37°C, and β-galactosidase assays were performed according to Miller (37).

### Catabolite repression.

The PA14 wild type carrying pJAM22 was grown overnight at 37°C in MOPS with 20 mM pyruvate, 5 mM glucose, and 20 µg·ml^−1^ gentamicin. Cells were collected by centrifugation, washed, resuspended in MOPS, and then inoculated into MOPS with 20 mM pyruvate and 20 µg·ml^−1^ gentamicin at a final OD_600_ of 0.05. All samples had a 1 mM concentration of the inducting compound carnitine, except for the noninducing control (pyruvate alone). Catabolite repression samples had glucose at 2, 4, or 10 mM or had glycine betaine at 20 or 40 mM. Cultures were induced for 4 h at 37°C, and β-galactosidase assays were performed according to Miller (37).

### EMSAs.

To determine binding of MBP-CdhR or MBP-GbdR to promoters, EMSAs were performed as previously described (17). Briefly, *caiX*, *dhc*, *cbcX*, *cdhR*, or *cdhR* mutant promoter DNA fragments were made by PCR amplification from PA14 genomic DNA template with primers listed in the EMSA primer section in Table 2 and verified by sequencing. The DNA probes were dialyzed in 1/4 Tris-EDTA (TE) on a 0.025-µm-pore filter for 20 min. EMSAs were conducted following the LightShift chemiluminescent EMSA kit instructions (Pierce) as modified by Hampel et al. (17) with labeled probes used at 1 fmol·µl^−1^ and unlabeled competitor at 600 fmol·µl^−1^. Samples were electrophoresed on 5% Tris-borate-EDTA (TBE) nondenaturing gels, transferred to BioDyne-B nylon membrane (Thermo), and detected using the Thermo chemiluminescent nucleic acid detection module per the manufacturer’s instructions.

### DNase I footprinting.

DNase I footprinting was performed as described previously (17). Briefly, the target DNA was made by labeling the 5′ end of either PA5388promR or PA5388promF2 primer with ^32^P using T4 polynucleotide kinase and [γ-^32^P]ATP and amplifying the PA14 *caiX* upstream region. The radiolabeled PCR product was purified from a 5% polyacrylamide Tris-borate-EDTA gel. The MBP-CdhR footprinting assay was conducted as in Brenowitz et al. (38), as modified in the study by Hampel et al. (17).

### Fluorescence microscopy.

*P. aeruginosa* PAO1 WT, Δ*cdhR*, and Δ*gbdR* strains with the divergent fluorescent reporter *PA5388-PA5389* intergenic region CFPYFP-DR2 (pJAM86) at the *att*Tn7 site, were grown overnight in MOPS with 20 mM pyruvate and 5 mM glucose at 37°C. Cells were collected by centrifugation, washed, and resuspended in MOPS to an OD_600_ of 1.0. A 1/20 dilution was made, and 1 µl was placed on the center of an agar pad (1.5% low-melting-point agarose, MOPS medium with 20 mM pyruvate, and with or without 1 mM carnitine). The agar pad was placed cell side down on a 50-mm glass bottom cell culture dish (Warner Instruments). Samples were imaged on a Nikon Ti-E every 10 min for 6 h at 32°C. Image stacks were imported into Fiji 2.0.0-rc-29/1.49 s (39) using the Bio-Formats importer 5.1.1 (40). A rolling variance filter on the contrast channel was used to define background areas, and after an additional threshold was applied, the “find maxima” macro was used to define cell areas. Using masks from the contrast channel, mean pixel intensity measurements were taken from the YFP channel. A framewise background intensity correction was performed for each cell using R 3.2.0 (41).
